# Diffuse Mesenteric and Bowel Angiodysplasia: A Case Report

**DOI:** 10.7759/cureus.76039

**Published:** 2024-12-19

**Authors:** Muna S Gumaa Albashari, Salsabil K Abbad, Mohammad H Turki Al helal, Bader N Almansour

**Affiliations:** 1 College of Medicine, Alfaisal University College of Medicine, Riyadh, SAU; 2 Faculty of Medicine, Elrazi University, Khartoum, SDN; 3 General Surgery, King Saud Medical City, Riyadh, SAU

**Keywords:** hematochezia, hereditary bleeding disorder, intestinal angiodysplasia, intraoperative endoscopy, malena, massive lower gi bleeding, small bowel resection

## Abstract

Angiodysplasia is one of the causes of recurrent episodes of lower gastrointestinal (GI) bleeding. Angiodysplasia could be associated with few lesions or multiple diffuse lesions, causing diversity in the clinical presentation of such patients. We report a case of a 19-year-old male presenting with life-threatening gastrointestinal bleeding due to diffuse angiodysplasia of the bowel extending from the jejunum to the sigmoid colon and requiring multiple investigations and management.

## Introduction

Gastrointestinal (GI) bleeding is any bleeding that originates in the gastrointestinal tract and extends from the mouth to the anus. It can be further divided into upper and lower gastrointestinal bleeding, differentiated by the ligament of Treitz. Lower GI bleeding is bleeding distal to the ligament of Treitz and usually presents as hematochezia or melena. This type of bleeding accounts for 20% to 30% of all patients with major GI bleeding. Diverticulosis is the most common cause of lower gastrointestinal bleeding [1]. Another possible cause is angiodysplasia, which is considered the most common vascular anomaly in the GI tract [2,3].

Angiodysplasia is an acquired, abnormal, dilated, and tortuous malformation of small blood vessels in the intestinal mucosa and submucosa. The ascending colon is considered the most common site, followed by the terminal ileum. These abnormal vessels are usually lined only with endothelial cells with little or no smooth muscle. Angiodysplasia tends to occur in people over the age of 60, and the exact prevalence of angiodysplasia is unknown [3,4]. Advances in imaging and endoscopy have clearly increased the detection rate of angiodysplasia [2]. Angiodysplasia presents with diverse clinical features of bleeding, ranging from signs of iron deficiency anemia and occult blood in the stool to massive hemorrhage [4]. Our case illustrates diffuse gastrointestinal angiodysplasia as a source of life-threatening gastrointestinal bleeding.

## Case presentation

A 19-year-old male with a possible bleeding disorder presented to the emergency department with nausea and vomiting of food content for two days associated with generalized abdominal pain. His detailed history is significant for recurrent episodes of melena, recurrent epistaxis, and bleeding at circumcision. The patient had no previous surgeries, is not on any medications, and does not use illicit drugs or smoke. He has a positive family history of bleeding disorders in his sister and aunt. The patient was afebrile and tachycardic with a heart rate of 110 beats per minute (BPM), blood pressure of 129/64 mmHg, respiratory rate of 20 breaths per minute, and oxygen saturation of 98% on room air. Physical examination was remarkable for generalized pallor and diffuse abdominal pain with no rigidity or rebound tenderness. A week earlier, he had been admitted to the hematology department for lower GI bleeding and was treated conservatively with a blood transfusion. During this visit, his platelet count and coagulation profile were within normal levels. Since his last visit, his hemoglobin level has dropped from 9.2 mg/dl to 5.7 mg/dl. Peripheral smear showed severe normocytic normochromic anemia with unremarkable WBC and platelets, and the direct Coombs test was negative. Other laboratory tests were all unremarkable. The patient received 24 units of packed red blood cells (PRBC), fresh frozen plasma (FFP), platelets, and omeprazole infusions during his stay at the hospital. He also received octreotide, thalidomide, tranexamic acid, and desmopressin without improvement and persistent episodes of melena.

**Figure 1:**
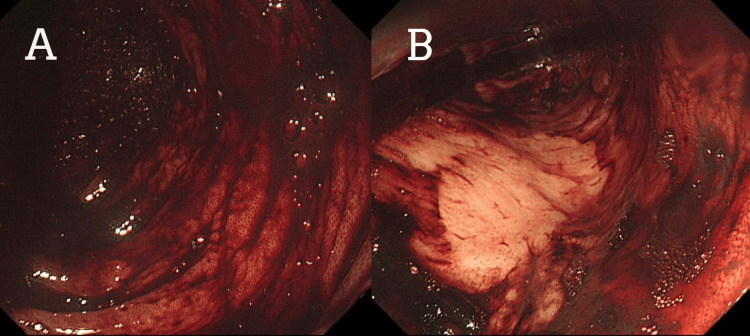
Colonoscopy: showing fresh blood and melena in the descending colon (A) and the sigmoid colon (B).

**Figure 2:**
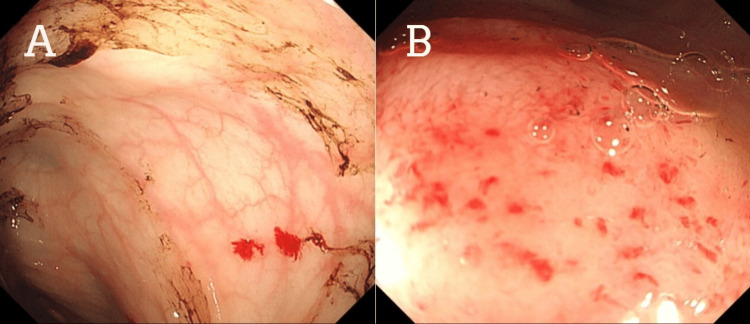
Colonoscopy: multiple angiodysplastic lesions in the sigmoid colon (A) and the terminal ileum (B).

**Figure 3:**
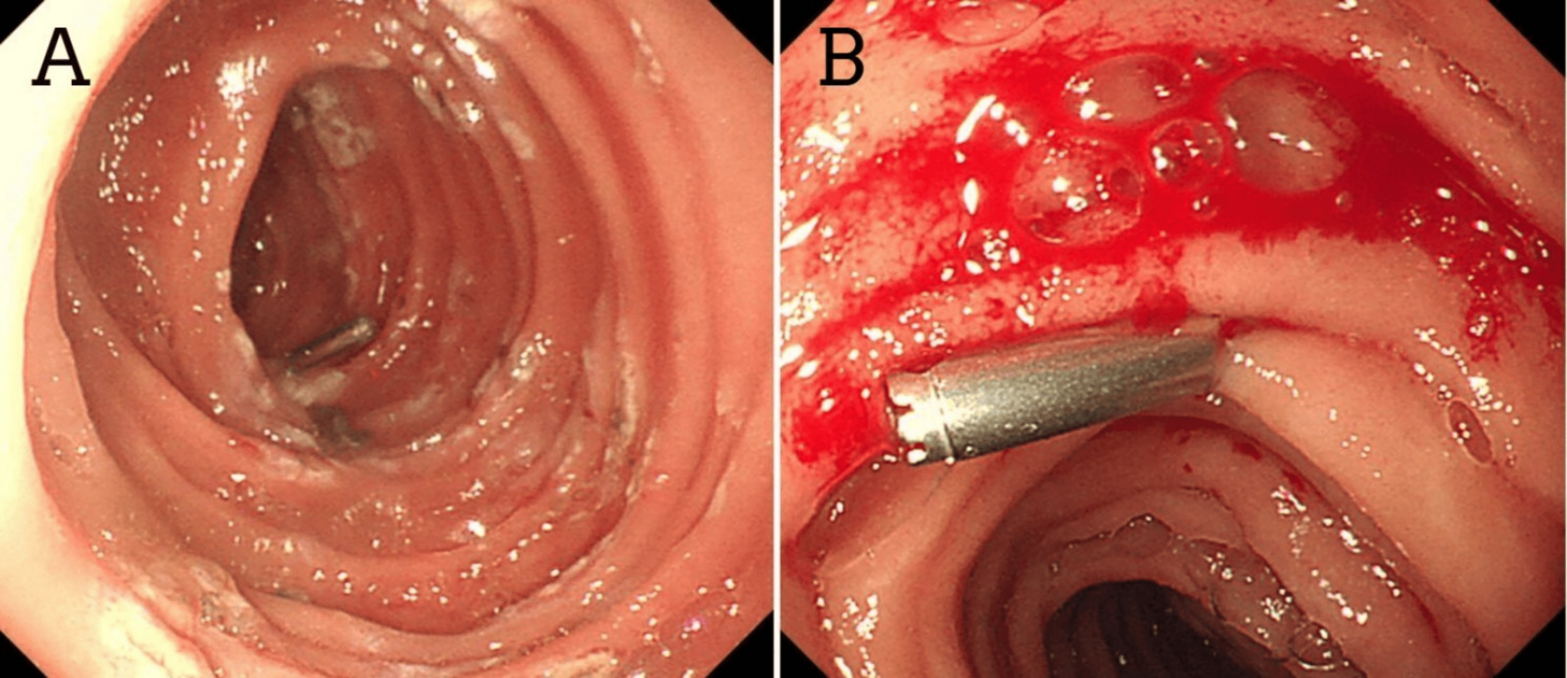
Enteroscopy: (A) and (B) coagulation using argon plasma with one hemostatic clip placed for marking.

Multiple endoscopies were done on the patient. Upper GI endoscopy was unremarkable. A colonoscopy showed red blood mixed with melena in the rectum, the sigmoid colon, the descending colon, and the splenic flexure (Figure 1). Tc-99m was performed, and brisk bleeding was seen in the descending colon. Meckel's scan was negative. Repeated colonoscopy with better bowel preparation revealed multiple small angiodysplastic lesions with stigmata of recent bleeding in the sigmoid colon (Figure 2). One small angiodysplasia in the cecum was found, and argon plasma coagulation (APC) was performed. The terminal ileum contained multiple small angiodysplastic lesions without bleeding (Figure 2). The scope was advanced up to 70 cm from the ileocecal valve with few nonbleeding angiodysplasia seen. A capsule endoscopy was performed with fresh blood seen, but no clear source of bleeding was identified. During a repeated endoscopy, red blood was aspirated, and multiple 5 mm angiodysplastic lesions with active bleeding were found in the jejunum. Coagulation for hemostasis using argon plasma was performed, and one hemostatic clip was successfully placed for marking (Figure 3).

**Figure 4:**
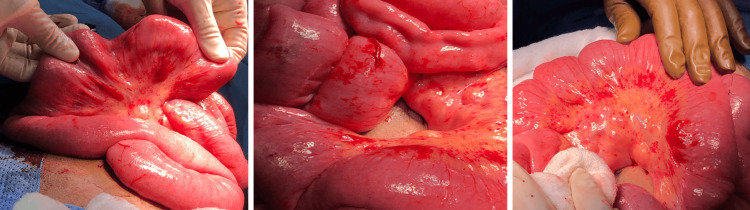
Intraoperative: diffuse mesenteric and bowel angiodysplasia.

Due to the patient's low hemoglobin, he was transferred to the ICU. The patient was hypotensive and was given intravenous fluid, ceftriaxone, and more than 40 units of blood. As a consequence of the persistent bleeding despite the previous medical and endoscopic management, the patient was referred for laparotomy with intraoperative endoscopy (IOE), small bowel resection, and anastomosis. During the procedure, the small bowel was filled with blood and melena. Intraoperative endoscopy was performed, which revealed an area of angiodysplasia 60 cm away from the duodenojejunal junction, and another bleeding angiodysplasia 50 cm distal was also found. The whole bowel from the duodenojejunal junction to the ileocecal valve showed multiple nonbleeding angiodysplastic lesions (Figure 4), and resection of a 70 cm segment from the distal jejunum-proximal ileum with anastomosis was done. Histopathology of the resected segment showed submucosal and mesenteric clusters of tortuous, dilated, and thin-walled blood vessels consistent with arteriovenous malformation. Furthermore, multiple patchy mucosal erosions and ulceration with granulation tissue were seen.

The patient was returned to the ICU, where his condition improved significantly. Blood products and tranexamic acid were administered as needed. The patient was then extubated. He stopped experiencing hematochezia and gradually reduced his melena until his stool returned to normal. Finally, he was transferred to the ward and then discharged home.

## Discussion

Gastrointestinal angiodysplasia is primarily associated with recurrent episodes of lower GI bleeding. In our case, the patient was found to have diffuse angiodysplasia of the gastrointestinal tract with hematochezia, melena, and a sharp drop in hemoglobin, requiring blood transfusion and surgical intervention [2]. GI angiodysplasia can be classified as single, multiple, or diffuse angiodysplasia (defined as >10 lesions). 40-60% of cases have multiple lesions in the same area, and 20% have lesions in other areas of the gastrointestinal tract [5]. In this case, the patient had diffuse gastrointestinal angiodysplasia extending from the jejunum to the sigmoid colon and affecting the mesentery. Furthermore, gastrointestinal bleeding is classified into massive bleeding, moderate bleeding, and occult bleeding. Massive bleeding, such as that seen in this patient, is often associated with angiodysplasia but is usually associated with the presence of comorbidities and advanced age. In a multicenter study, the majority of patients with gastrointestinal bleeding due to angiodysplasia were elderly. In addition, comorbidities associated with the development of gastrointestinal bleeding include aortic stenosis, chronic anticoagulant therapy, end-stage renal disease, and congestive heart failure [2], which were all negative in our patient, except for the presence of a not-yet-diagnosed bleeding disorder.

Given the positive history of recurrent bleeding and post-circumcision bleeding, it is highly likely that a bleeding disorder contributed to this patient's condition. The most common bleeding disorder associated with angiodysplasia of the gastrointestinal tract is von Willebrand disease (VWD). VWD is an autosomal inherited disorder. 1% of the population is affected, and only 0.1% are symptomatic. The prevalence of patients with VWD with GI bleeding is 2.5 times higher than in other populations, and 36.5% of VWD patients eventually develop GI angiodysplasia [6]. Although GI angiodysplasia is common in VWD patients, where they commonly present with melena, as in our case, it was found that the median age of patients with angiodysplasia was 65 years [7]. Other cases of VWD patients presented with various symptoms, including early epistaxis, prolonged bleeding, and menorrhagia, followed by gastrointestinal bleeding at age 40 years and older [8-11]. Other possible diagnoses are Glanzmann's thrombasthenia (GT) and Bernard-Soulier syndrome. GT is an autosomal recessive disorder associated with a defect in GIIb/IIIa. It is estimated that one in 1,000,000 suffers from Glanzmann's thrombasthenia. Patients usually present with mucocutaneous bleeding in the form of epistaxis and bruising [12]. GI bleeding can occur, leading to severe bleeding in 20% of the cases [13]. Bernard-Soulier syndrome is also an autosomal recessive platelet disorder with a defect in GPIbIX/V, causing similar mucocutaneous bleeding to GT. Both disorders are rare but have a higher occurrence in families with consanguinity [14].

Obtaining a good history and physical examination, along with laboratory investigation and imaging, could be essential in such cases. Diagnosis can be made using upper endoscopy, which is followed by colonoscopy. If both are inconclusive, a video capsule endoscopy can be done. Other alternative investigations for GI bleeding include CTA, catheter angiography, and radionuclide imaging using technetium (99mTc) [1]. Initial upper and lower gastrointestinal endoscopy and capsule endoscopy were inconclusive, as noted in this case. This may be due to poor preparation of the gastrointestinal tract or the presence of active bleeding. Thus, repeated endoscopies were required to confirm the presence of angiodysplasia.

Angiodysplasia can be treated endoscopically, pharmacologically, and in severe cases, surgically [15]. Endoscopy is usually recommended within 24 hours of symptom onset and can prevent some adverse effects, such as the risk of rebleeding [16]. Balloon, push, or endocuff enteroscopy can be used for treatment. If there is active bleeding, cauterizers or clips can be used [15]. Argon coagulation or bipolar electrocoagulation should be used when a single or few angiodysplastic lesions can be accessed using endoscopic therapy. Additionally, laser endoscopy is also a very effective option but carries the risk of intestinal perforation [17]. Likewise, argon coagulation was done for this patient, but bleeding and melena persisted. Surgery is the last resort in case of unresponsiveness to resuscitation, hemodynamic instability, or the need for more than 6 units of blood in less than a day [1]. In our case, the patient received more than 40 units of blood products with persistent bleeding. Therefore, surgical resection was required. IOE from an enterostomy site was used. With a diagnostic rate of 60-88%, intraoperative endoscopy is a good option to perform during surgery. IOE helps visualize the small intestine and identify lesions and areas that may not be accessible with conventional endoscopy [18]. Although intraoperative enteroscopy followed by surgical intestinal resection is an option, the development of other vascular dysplastic lesions eventually occurs, making surgical options less desirable [6].

Medical management is an option in the treatment of angiodysplasia. Somatostatin analogs, such as lanreotide, are pharmacological options that reduce the need for iron supplementation and blood transfusions and reduce the recurrence of bleeding both in the short and long term. However, other studies have found lanreotide to be ineffective, expensive, and of unpredictable efficacy [15]. Another pharmacological therapy is thalidomide. It helps inhibit the vascular endothelial growth factor (VEGF), which contributes to an increased risk of gastrointestinal bleeding [19,20]. Additionally, tranexamic acid acts as an antifibrinolytic agent, reducing fibrinolytic activity that may contribute to the development of gastrointestinal angiodysplasia [20]. Other pharmacological treatments, such as statins and steroids, can also be used [6]. For patients with VWD specifically, desmopressin can be used for the management of bleeding. Von Willebrand factor concentrate is effective by 94.9% in cases of acute bleeding and serves as an effective prophylactic method to prevent bleeding (84.6%) [17]. In our patient, multiple pharmacological options were used with no improvement. Other multiple case reports have identified a variety of responses to pharmacological management as well, emphasizing the individualization of management in patients with angiodysplasia [8-11]. Therefore, the need to assess the response of such patients to the available methods of management is crucial to optimizing their care.

## Conclusions

Angiodysplasia is one of the main causes of massive GI bleeding. This is especially true if the patient is suspected of having bleeding disorders. To diagnose and treat gastrointestinal angiodysplasia early and prevent the recurrence of bleeding, a high index of suspicion, along with proper investigation and management, is required. Multiple management options are available to eliminate angiodysplasia and improve the symptoms, but their effectiveness and degree of patient response vary. Although surgical management of angiodysplasia is associated with a risk of recurrence and is considered a last resort, it becomes necessary when bleeding persists despite medical and endoscopic interventions. This was a case of diffuse angiodysplasia that improved after surgical resection assisted by intraoperative endoscopy.
